# Modified Titanium Surface-Mediated Effects on Human Bone Marrow Stromal Cell Response

**DOI:** 10.3390/ma6125533

**Published:** 2013-11-28

**Authors:** Amol Chaudhari, Joke Duyck, Annabel Braem, Jozef Vleugels, Hervé Petite, Delphine Logeart-Avramoglou, Ignace Naert, Johan A. Martens, Katleen Vandamme

**Affiliations:** 1 BIOMAT Research Cluster, Department of Oral Health Sciences and Prosthetic Dentistry, KU Leuven and University Hospitals Leuven, Kapucijnenvoer 7 box 7001, Leuven 3000, Belgium; E-Mails: Amol.Chaudhari1911@gmail.com (A.C.); Joke.Duyck@uzleuven.be (J.D.); Ignace.Naert@uzleuven.be (I.N.); 2 Department of Metallurgy and Materials Engineering (MTM), KU Leuven, Kasteelpark Arenberg 44 box 2450, Heverlee 3001, Belgium; E-Mails: annabel.braem@mtm.kuleuven.be (A.B.); jozef.vleugels@mtm.kuleuven.be (J.V.); 3 Laboratory of Bioengineering and Biomechanics for Bone Articulation, Faculty of Medicine, University Paris Diderot, 10 Avenue de Verdun, Paris 75010, France; E-Mails: Herve.Petite@univ-paris-diderot.fr (H.P.); Delphine.Logeart@univ-paris-diderot.fr (D.L.-A.); 4 Center of Surface Chemistry and Catalysis, KU Leuven, Kasteelpark Arenberg 23 box 2461, Heverlee 3001, Belgium; E-Mail: johan.martens@biw.kuleuven.be

**Keywords:** titanium, surface coating, human bone marrow stromal cells, *in vitro* cytocompatibility, osteogenic differentiation, osseointegration

## Abstract

Surface modification of titanium implants is used to enhance osseointegration. The study objective was to evaluate five modified titanium surfaces in terms of cytocompatibility and pro-osteogenic/pro-angiogenic properties for human mesenchymal stromal cells: amorphous microporous silica (AMS), bone morphogenetic protein-2 immobilized on AMS (AMS + BMP), bio-active glass (BAG) and two titanium coatings with different porosity (T1; T2). Four surfaces served as controls: uncoated Ti (Ti), Ti functionalized with BMP-2 (Ti + BMP), Ti surface with a thickened titanium oxide layer (TiO_2_) and a tissue culture polystyrene surface (TCPS). The proliferation of eGFP-fLuc (enhanced green fluorescence protein-firefly luciferase) transfected cells was tracked non-invasively by fluorescence microscopy and bio-luminescence imaging. The implant surface-mediated effects on cell differentiation potential was tracked by determination of osteogenic and angiogenic parameters [alkaline phosphatase (ALP); osteocalcin (OC); osteoprotegerin (OPG); vascular endothelial growth factor-A (VEGF-A)]. Unrestrained cell proliferation was observed on (un)functionalized Ti and AMS surfaces, whereas BAG and porous titanium coatings T1 and T2 did not support cell proliferation. An important pro-osteogenic and pro-angiogenic potential of the AMS + BMP surface was observed. In contrast, coating the Ti surface with BMP did not affect the osteogenic differentiation of the progenitor cells. A significantly slower BMP-2 release from AMS compared to Ti supports these findings. In the unfunctionalized state, Ti was found to be superior to AMS in terms of OPG and VEGF-A production. AMS is suggested to be a promising implant coating material for bioactive agents delivery.

## 1. Introduction

Continuous progress in understanding the concept of osseointegration has lead clinicians to achieve high success rates in implant therapy [1,2,3]. Nevertheless, the conditions of limited bone healing potential (such as diabetes, smoking, irradiated bone, grafted bone) and of immediate implant loading may lead to increased implant failure rates [4,5,6]. In order to achieve successful implant osseointegration in those situations, increasing interest has emerged to find new ways to improve the quality and kinetics of osseointegration, by means of biomechanical interventions and implant macro- and micro-design optimization, amongst others [7,8,9]. Implant surface modifications stimulating the recruitment of premature osteogenic cells may lead to an enhanced peri-implant bone formation and implant osseointegration [10]. It is, however, difficult to predict cell behavior, even on a surface of well-known properties [11]. Experimental evidence showed that osteoprogenitor cells have a higher affinity for implants with rough surfaces (macro-, micro- and nano-level topographies) [12] and for functionalized surfaces [13] compared to polished and non-functionalized ones. It is therefore desirable to apply implant surface modification techniques that evoke cell stimulation towards bone regeneration.

In the present study, altering the implant surface properties for achieving accelerated differentiation of progenitor cells into the osteogenic lineage was aimed for. Five experimental surfaces were tested. One of the surface alterations under investigation was the coating of titanium (Ti) with amorphous microporous silica (AMS) [14]. Silica (SiO_2_)-based implant surfaces are attractive for bone regeneration applications, owing to their biocompatible nature, nanostructure and potential use as vehicle for growth factor delivery at the implant site [15,16,17]. Moreover, nano-structured surfaces may stimulate cells through initial surface protein interactions [13]. Based on the evidence that AMS is suitable for incorporation and controlled release of bioactive molecules [14,18,19], this material was further tested as the delivery vehicle for bone morphogenetic protein 2 (BMP-2), the most potent osteoinductive proteins of the BMP group [20]. The third surface modification was prepared by use of a bio-active glass coating (BAG) [21]. BAG is known for its bone formation capability at an endosseous implant surface, with a resulting faster osseointegration [22]. Ionic components released from BAG material in the biological environment, such as Ca^2+^ and PO_4_^3−^, promote osteoconduction by forming a calcium phosphate-like apatite layer onto the implant surface [23]. The last two surfaces under consideration were porous coatings of particulate titanium. It is known that such surfaces with roughness in the micrometer range and porosities above 50% assist the mechanical interlocking of the implant with the host tissues and, thereby, the ultimate bone regeneration [24,25,26]. In order to combine the biocompatible nature of the titanium surface with the osteoinductive properties of a porous surface, porous coatings of particulate Ti were made on the Ti substrate. A simple technique of electrophoretic deposition of TiH_2_ via suspension and emulsion followed by sintering was used to produce such coatings [27,28].

Altogether, the biological behavior of five experimental surfaces was evaluated *in vitro*: (1) AMS-coated Ti surface; (2) BMP-2 adsorbed onto the AMS-coated Ti surface; (3) BAG-coated Ti surface; and (4) and (5) two porous coatings of pure, particulate Ti with a rough topography and varying 3D morphology. Four surfaces served as controls: uncoated Ti surface (Ti), Ti surface with adsorbed BMP-2 (Ti + BMP), Ti surface with a thickened titanium oxide layer (TiO_2_) and tissue culture polystyrene surface (TCPS). The aim of the present work was to evaluate the possible beneficial effects of the experimental surfaces on cell proliferation and differentiation towards the osteogenic lineage. To do so, human mesenchymal stromal cells (hMSCs) were selected.

## 2. Results and Discussion

### 2.1. Surface Characterization

A wide range of hydrophilicity for the different surfaces was observed (Table 1). The AMS surface displayed a significantly smaller contact angle (CA) than the uncoated Ti surface. Owing to adsorption of BMP-2 onto Ti and AMS, the surfaces became very hydrophilic, and the CA could no longer be measured. Thickening of the oxide layer of the Ti surface (TiO_2_) and application of the BAG coating significantly reduced the CA to 45.10° ± 0.67° and 0°, respectively. T1 and T2, on the other hand, had a significantly higher CA compared to the control Ti surface.

The S_a_ (the arithmetic mean of absolute values of the surface departures from the mean plane) values of all surfaces, except TiO_2_, were significantly higher compared to the control Ti surface (Table 1). Furthermore, the surfaces, T1 and T2, significantly differed mutually in S_a_ values. Significantly higher S_tr_ (the measure of spatial isotropy or directionality of the surface texture) values were observed for BAG, T1 and T2, while AMS, Ti + BMP and AMS + BMP surfaces had lower S_tr_ values compared to uncoated Ti surface. A change in the surface area as measured by S_dr_ (the percentage of additional surface area due to the surface modification as compared to measurement of an ideal plane) was significantly higher for AMS, Ti + BMP, AMS + BMP, BAG, T1 and T2 compared to uncoated Ti surface. It is suggested that the considerable difference in topographic parameters of the BAG, T1 and T2 surfaces (and of the TiO_2_ surface, though to a minor extent) compared with the (un)coated Ti and AMS surfaces are responsible for the poor cytocompatibility of these surfaces (*cf*. Section 2.2).

**Table 1:** Characterization of the modified titanium surfaces. AMS, amorphous microporous silica; BMP, bone morphogenetic protein; BAG, bio-active glass.

Group	Contact Angle (°)	Topography parameter		
		S_a_ (µm)	S_tr_	S_dr_ (%)
**Ti**	75.53 ± 0.51	0.29 ± 0.005	0.54 ± 0.02	0.35 ± 0.01
**AMS**	36.56 ± 0.21 *	0.61 ± 0.01 *	0.32 ± 0.06 *	0.91 ± 0.03 *
**Ti + BMP**	≈0	0.61 ± 0.02 *	0.24 ± 0.03 *	1.58 ± 0.11 *
**AMS + BMP**	≈0	0.64 ± 0.02 *	0.25 ± 0.03 *	1.68 ± 0.10 *
**TiO_2_**	45.10 ± 0.67 *	0.31 ± 0.01	0.53 ± 0.05	0.37 ± 0.01
**BAG**	≈0	3.25 ± 0.13 *	0.79 ± 0.02 *	97.23 ± 7.53 *
**T1**	99.22 ± 0.51 *	7.86 ± 0.21 *^,†^	0.70 ± 0.03 *	102.50 ± 6.26 *^,†^
**T2**	101.27 ± 0.32 *	9.06 ± 0.21 *^,†^	0.72 ± 0.03 *	188.98 ± 6.81 *^,†^

S_a_, arithmetic mean of absolute values of the surface departures from the mean plane; S_tr_, measure of spatial isotropy or directionality of the surface texture; S_dr_, percentage of additional surface area due to the surface modification as compared to measurement of an ideal plane; * significantly different from the uncoated Ti surface; ^†^ significant differences among the groups.

**Figure 1:**
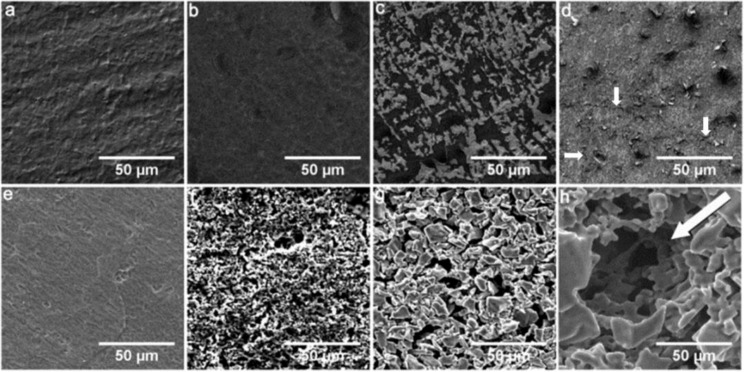
Qualitative SEM analysis of surface topography of the modified titanium surfaces. (**a**) Ti; (**b**) AMS; (**c**) Ti + BMP; (**d**) AMS + BMP; (**e**) TiO_2_; (**f**) BAG; (**g**) T1; and (**h**) T2. The arrow in figure (**h**) shows a typical hole created by the use of emulsion-based electrophoretic deposition of TiH_2_.

Qualitative analysis by scanning electron microscopy (SEM) suggested that the AMS surface is a rather uniform coating. The Ti + BMP coating surface was similar to the Ti surface, except for the white spots (Figure 1a–c). Some cracks on the AMS surface after BMP-2 adsorption could be observed, as shown in Figure 1d (as shown by the arrows). No visual change in the surface topography was noticed for TiO_2_ (Figure 1e). Deposition of BAG particles followed by sintering created randomly spread rough surface structures (Figure 1f). Rough surfaces were observed after the application of T1 and T2 coatings compared to uncoated Ti (Figure 1g,h *versus* 1a). Highly porous surfaces of T1 and T2 mutually differed by the presence of additional spherical holes of ~50 µm for T2. The differences in surface and inner morphology between T1 and T2 surfaces were attributed to the difference in medium (suspension *versus* emulsion) used for the electrophoretic deposition of TiH_2_ [28].

### 2.2. Proliferation of hMSC on Modified Ti Surfaces

**Figure 2:**
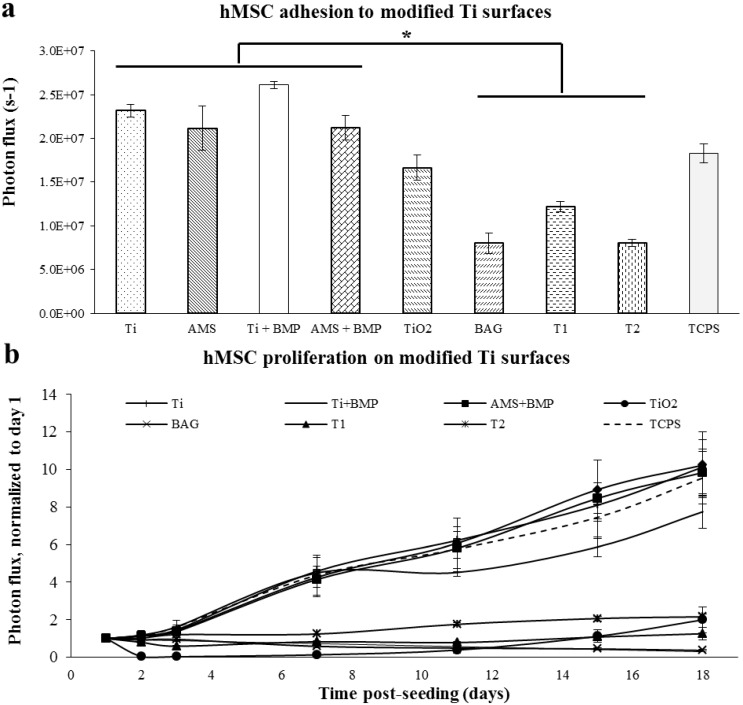
Adhesion of human mesenchymal stromal cells (hMSCs) at 24 h post-seeding (**a**) and proliferation (**b**) on the modified Ti surfaces. The experiment was carried out for 18 days; *n* = 3 for each data point; the results are the mean ± SEM. Statistically significant differences (* *p* < 0.02) were analyzed for each surface compared to the others. TCPS, tissue culture polystyrene surface.

The hMSC proliferation on modified Ti surfaces was monitored non-invasively by bioluminescence imaging (BLI). A significantly enhanced cell adhesion was noted for Ti, AMS, Ti + BMP and AMS + BMP compared to BAG, T1 and T2 surfaces (Figure 2a). Concerning the cell proliferation (Figure 2b), a steady and almost identical increase in the BLI signal was observed for Ti, AMS, Ti + BMP and AMS + BMP along with TCPS.

At day 18 post-seeding, a *circa* 10-fold increase in cellular coverage for all these surfaces was observed, significantly differing from the cell behavior in groups TiO_2_, BAG, T1 and T2. The latter surface groups could not support cell proliferation. The structure of T1 and T2 coatings resembles that of titanium plasma sprayed coatings [27,28], with a high porosity (>50%) (similar to the porosity of bone), thereby providing considerable interlocking strength across the bone-implant interface. However, according to animal studies, the effect of the properties of porous Ti coatings on implant osseointegration is still not elucidated [29,30,31,32,33]. Although hydrophilic surfaces are generally considered to promote cell attachment, the T1 and T2 surfaces may not have promoted cell adhesion, because of the determining role of the surface chemistry [34,35]. Therefore, in addition to surface wettability analysis, chemical data are required for a better understanding of the cell attachment behavior. Furthermore, although BAG is osteoinductive in the implant setting, owing to the release of Ca^2+^ and PO_4_^3−^, as illustrated by the authors’ group *in vivo* [36], the highly alkaline environment created by the BAG coatings may have been responsible for its toxic effect on the cells.

**Figure 3:**
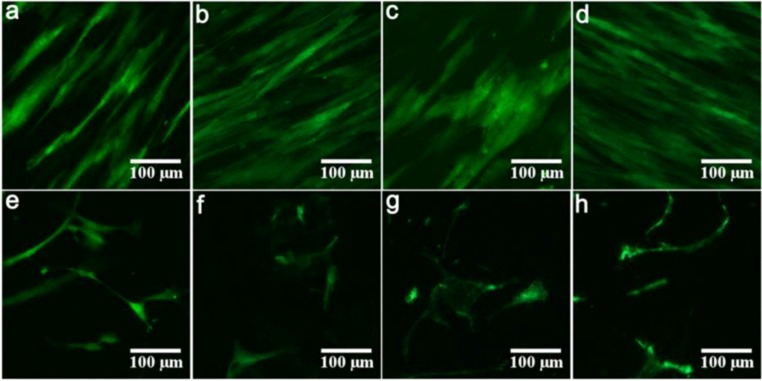
Representative fluorescence microscopy images of hMSCs cultured for 18 days on Ti (**a**); AMS (**b**); Ti + BMP (**c**); AMS + BMP (**d**); TiO2 (**e**); BAG (**f**); T1 (**g**) and T2 (**h**), respectively.

At the endpoint of the experiment, fluorescence microscopy revealed a well-aligned confluent cell layer on Ti, AMS, Ti + BMP and AMS + BMP, whereas few and apoptotic cells were observed on the TiO_2_, BAG, T1 and T2 surfaces (Figure 3a–h).

### 2.3. Differentiation of hMSC on Modified Ti Surfaces

As revealed by the results of the proliferation experiment, TiO_2_, BAG, T1 and T2 surfaces did not support cell adhesion and proliferation. Therefore, hMSC differentiation experiments were carried out only for the surfaces, AMS and AMS + BMP, along with the respective control surfaces (Ti, Ti + BMP and TCPS).

#### 2.3.1. Alkaline Phosphatase Activity

**Figure 4:**
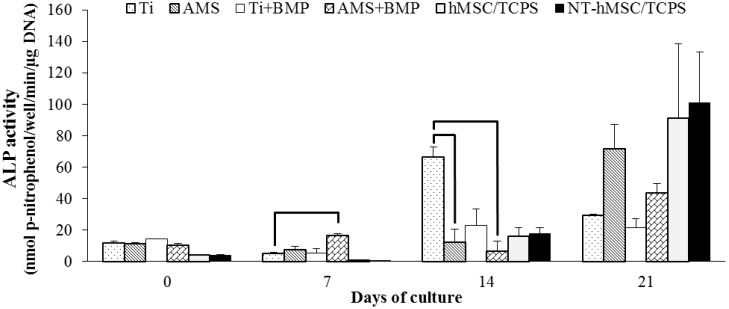
Alkaline phosphatase (ALP) activity of hMSCs cultured on modified Ti surfaces. *n* = 2 for each data point; the experiment was performed in duplicate. The results are the mean ± SD; statistically significant differences between the surface groups with *p* < 0.05 are shown by horizontal bars.

Alkaline phosphatase (ALP) activity results shown in Figure 4 are indicative of the osteogenic differentiation potential of the cell layer and of the surface-mediated effect herein. Up to seven days of osteogenic induction, higher ALP levels were observed for all experimental surface groups compared to the cell groups (*i.e*., the disc-free wells with cells seeded on TCPS). At the seven-day time point, the highest ALP activity was noted for the AMS + BMP group and significantly differed from the uncoated Ti surface, suggesting the potential of the former surface to induce a faster onset of hMSCs osteogenic differentiation. At day 14 of osteogenic induction, however, the ALP activity results revealed that the Ti surface significantly enhanced the further direction of the cells into the osteogenic pathway compared to the (un)functionalized AMS surface. Furthermore, coating of Ti with BMP did not affect the cell differentiation behavior, compared with the uncoated surface. At the 21-day endpoint of the osteogenic induction, statistical analysis revealed no differences between the surface groups. Progressively increasing ALP activity levels for the cells on TCPS display a good positive control and suggest an uncomplicated osteogenic differentiation of the cells. Besides, no differences in ALP activity of non-transfected-hMSCs and eGFP-fLuc (enhanced green fluorescence protein-firefly luciferase)-hMSCs were noted, suggesting no adverse effect of cell transfection on osteogenic differentiation.

#### 2.3.2. OC, OPG, VEGF-A and BMP-2 Release

**Figure 5:**
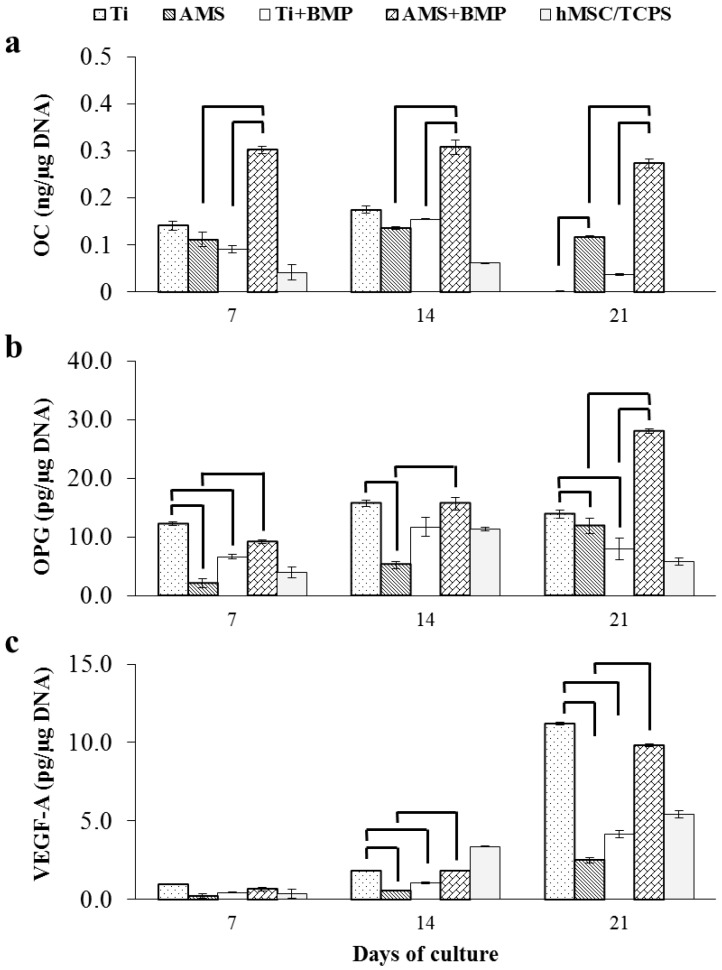
Osteogenic markers: Amount of osteocalcin (OC) (**a**); osteoprotegerin (OPG) (**b**); and vascular endothelial growth factor A (VEGF-A) (**c**) secreted into the culture medium by hMSCs cultured on modified Ti surfaces; *n* = 3 for each data point; the results are the mean ± SD. Statistically significant differences with *p* < 0.02 are shown by horizontal bars.

The amounts of osteocalcin (OC), osteoprotegerin (OPG) and vascular endothelial growth factor A (VEGF-A) secreted by the hMSCs into the culture medium after osteogenic induction for 21 days were measured by ELISA (Figure 5). In general, a higher OC secretion, a late marker for osteogenic differentiation, was observed for the surface groups compared to the cell group. Significantly higher OC release for AMS + BMP compared to non-functionalized AMS surface and the control surface with adsorbed BMP-2 (Ti + BMP) was observed. These differences were sustained during the entire assay duration. Unfunctionalized AMS coating, on the other hand, did not have an effect on osteoblastic differentiation compared to the control Ti. Therefore, it is suggested that BMP-2 adsorbed on AMS promoted hMSCs’ differentiation towards osteoblasts via its mediating effect on osteogenic markers, such as ALP and OC [37]. Furthermore, the superior (as quantified by the ALP “early cell differentiation” assay) support for osteoblastic differentiation of the unfunctionalized Ti surface compared to AMS could not be confirmed via the OC release recording, and this at all time points. Finally, it was observed (in line with ALP data) that BMP-2 adsorbed on the Ti surface did not affect the osteoblastic differentiation. It seems that the substrate influenced the activity of the adsorbed bioactive agent. For osteo-differentiation of progenitor cells, BMP-2 in free form is considered effective [38]. The specific release pattern of BMP-2 from the AMS surface might have positively influenced the osteogenic differentiation of hMSCs.

Osteoprotegerin (OPG) is an indicator of osteoclast inhibition during the cell differentiation process. Only small differences in OPG release over time were observed, except for a steady increase till day 21 for AMS + BMP. In analogy with the OC results, significantly higher OPG releases for AMS + BMP compared to the naked surface (AMS) were observed, revealing the potential for the inhibition of osteoclast formation by AMS + BMP during the process of osteogenic differentiation. Furthermore, significant differences between AMS + BMP and the control surface with adsorbed BMP-2 (Ti + BMP) could be found at day 21, though a similar trend was observed for other time points. Finally, in contrast to AMS + BMP *versus* AMS, functionalization of Ti with BMP-2 resulted in a decreased potential to impede osteoclastogenesis compared to the unfunctionalized Ti surface. Furthermore, this uncoated Ti displayed superior properties for the inhibition of osteoclast formation compared to AMS. From these observations, it is suggested that modification of the Ti surface is beneficial for impairing osteoclast development when BMP-2 is combined with AMS.

The onset of considerable amounts of VEGF-A for all groups was observed only after 14 days of osteogenic induction. Significantly higher VEGF-A release was observed for the control Ti surface compared to AMS and Ti + BMP. In line with OC and OPG results, VEGF-A release for AMS + BMP was significantly higher than unfunctionalized AMS surface and attained the same level as the control Ti after 21 days. Based on the compiled supernatant analyses, pro-osteogenic and pro-angiogenic properties for AMS + BMP are suggested.

**Figure 6:**
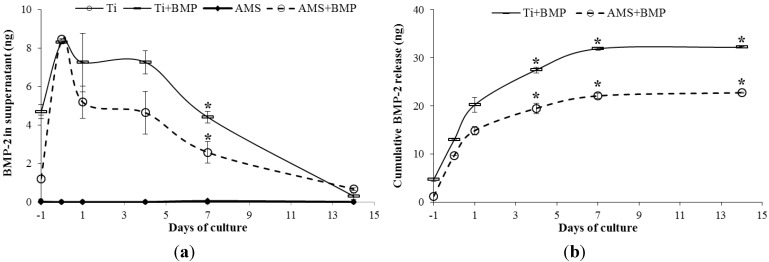
BMP-2 release profiles. (**a**) Amount of BMP-2 released into the cell culture medium from the surface of Ti + BMP and AMS + BMP. BMP-2 was not found in the supernatants of Ti and AMS; (**b**) Cumulative BMP-2 released from Ti and AMS surfaces with adsorbed BMP-2. The results are the mean ± SEM. The statistically significant differences are shown by horizontal bars; * *p* < 0.02.

Finally, the results of BMP-2 releases from Ti + BMP and AMS + BMP surfaces during cell differentiation are presented in Figure 6. The release profile was determined at six time points over a period of 14 days. Negligible amounts of secreted BMP-2 for the Ti and AMS groups were determined at all time points. In contrast, BMP-2 was found in the supernatants of the Ti + BMP and AMS + BMP groups. These findings suggest a minor contribution of endogenously produced BMP-2 to the total amount recorded. A significantly higher release of BMP-2 was noted at day 7 for Ti + BMP compared to AMS + BMP (Figure 6a). Cumulative release data representation confirms this difference in release profile between both groups (Figure 6b). Considering the amount of BMP-2 initially adsorbed onto the surfaces *versus* the amount of protein released after 14 days, an ongoing BMP-2 release during the entire cell differentiation experiment (and thereafter) can be expected. It is anticipated that this slow release pattern is advantageous for bone-biomaterial integration. Bone healing requires a certain period of time, relative to the bone turnover rate of specific animal species/humans and the continued availability of BMP at the implant surface, at a concentration anabolic for bone healing [39], which is beneficial for implant osseointegration.

## 3. Experimental Section

### 3.1. Titanium Discs as Substrate

A commercially pure Ti sheet (grade 2, Goodfellow, Huntingdon, UK) of 1 mm thickness was laser-cut into discs (Ø 15 mm). After ultrasonic cleaning in acetone (Acros Organics, Geel, Belgium) and washing in excess distilled water, the discs were acid-etched in a solution containing 4 vol % hydrofluoric acid (40%, Honeywell Riedel-de Haën, Heverlee, Belgium) and 20 vol % nitric acid (65%, ChemLab, Zedelgem, Belgium) at room temperature for 60 s. Ti discs were then washed again in distilled water and autoclave sterilized in separate inert containers. The titanium surface (Ti) is the substrate for the experimental coatings.

### 3.2. Titanium Surface Modification

#### 3.2.1. Amorphous Microporous Silica Coating

AMS was prepared according to Maier *et al*. [36]. Appropriate amounts of tetraethyl orthosilicate (TEOS, 98%; Acros Organics, Geel, Belgium), absolute ethanol (VWR, Haasrode, Belgium) and hydrochloric acid (HCl, 37%; ChemLab, Zedelgem, Belgium) were mixed to obtain a colloidal sol. The molar ratios of TEOS:ethanol:HCl:H_2_O were 1:3:1.74:6. A sol was obtained by stirring the mixture for 1 h at 250 rpm (Variomag Multipoint 15, Daytona Beach, FL, USA). The sol was then diluted 5-fold in absolute ethanol. Diluted sol was immediately spin-coated on the Ti disc surface using a spin-coating device (ERICHSEN GmbH & Co. KG, Hemer, Germany). Spin-coating was performed at 1500 rpm for 60 s. The coatings were dried at room temperature. Finally, calcination of the coatings was carried out by heating up to 65 °C for 5 h, followed by further heating till a final temperature of 300 °C. A heating rate of 0.1 °C/min^−1^ was maintained throughout the complete calcination procedure. The final temperature was sustained for 5 h, after which, coated surfaces were cooled to ambient temperature.

#### 3.2.2. BMP-2 Adsorption on AMS-Coating

Ten micrograms of BMP-2 (R&D Systems, Minneapolis, MN, USA) was reconstituted in 100 µL phosphate buffered saline (PBS) (pH 7.4, 0.01 M), as per the manufacturer’s instructions. Ten microliters of reconstituted protein (1 µg) was allowed to spread over the AMS-coated surface and to evaporate, so that exactly 1 µg of protein remained on the surface. In order to evaluate the potential of AMS coating as a delivery vehicle, BMP-2 was also adsorbed on an uncoated Ti surface. The 2 surfaces with adsorbed BMP-2 were designated as “AMS + BMP” and “Ti + BMP”. These surfaces were prepared under laminar flow just before the start of the cell culture experiments.

#### 3.2.3. Bio-Active Glass Coating

Preliminary experiments showed that BAG coatings were found to have better mechanical properties if made on a thickened titanium dioxide (TiO_2_) layer onto the Ti surface (results not shown). Therefore, the TiO_2_ layer was thickened by anodic oxidation in 1 M phosphoric acid (85%; Honeywell Riedel-de Haën, Heverlee, Belgium) using a controlled voltage power supply (MCN 1400-50, F.u.G. Elektronik GmbH, Rosenheim, Germany). On this surface, BAG coatings were applied by dipping the substrates for 120 s in 5 wt % absolute ethanol-based suspension of melt-derived BAG powder with a nominal composition of 50.1 wt % SiO_2_, 25.2 wt % CaO, 20.1 wt % Na_2_O and 4.6 wt % P_2_O_5_ and an average particle size of 1 µm. The samples were allowed to dry for 24 h and were sintered at 800 °C by resistive heating in vacuum (10^−6^ mbar). The cell biological response was evaluated for the BAG-coated surface, as well as for the uncoated, thickened TiO_2_ layer.

#### 3.2.4. Porous Ti Coatings (T1; T2)

Two types of porous coatings of pure Ti were made by electrophoretic deposition (EPD) of titanium hydride (TiH_2_) powder followed by dehydrogenation and sintering in vacuum. The first type of coating (T1) was applied as described previously by Braem *et al*. [34]. Briefly, TiH_2_ powder (grade P, Chemetall GmbH, Frankfurt (M), Germany) suspension in absolute ethanol (analytical grade, Prolabo, Haasrode, Belgium) was prepared using polyethyleneimine (PEI, 50 wt % in water; Sigma, Bornem, Belgium) as the charger and binder. The suspension was consolidated through EPD, using a controlled voltage power supply (MCN 1400-50, FUG, Rosenheim, Germany). The Ti substrate was vertically placed in a deposition cell and coupled as the cathode. Finally, the samples were dehydrogenated at 650 °C and sintered at 850 °C by resistive heating in vacuum (10^−6^ mbar). For the second porous Ti coating (T2), TiH_2_ powder stabilized emulsion was prepared by the procedure as used by Mattheys *et al*. [35]. For TiH_2_ powder deposition, cyclohexane (Prolabo, Haasrode, Belgium) was used as a dispersed phase. After the addition of the TiH_2_ suspension (as used for T1), an emulsion was consolidated through EPD and dehydrogenated and sintered in a similar manner as the T1 coating preparation.

#### 3.2.5. Control Surfaces

Besides the tissue culture polystyrene (TCPS) surface, uncoated Ti surface (Ti), uncoated Ti surface with adsorbed BMP-2 (Ti + BMP) and thickened TiO_2_ (TiO_2_) served as controls, as outlined above.

### 3.3. Surface Characterization

#### 3.3.1. Static Contact Angle Measurement

The static contact angle of water droplets was measured for all the surfaces at room temperature according to the sessile drop method using a video-based contact angle system (DSA10, Krüss, Hamburg, Germany). Mean contact angle values were determined by averaging 9 measurements obtained from 3 different samples in 3 different areas.

#### 3.3.2. Surface Topography

Quantitative 3D topographical analysis was performed using a scanning white light interferometer (Wyko NT 3300; Veeco Metrology Inc., Tucson, AZ, USA), employing a vertical scanning interferometer technique. Three samples per group were analyzed. A total of four equidistant locations were evaluated per surface, resulting in 12 measurements per group. Three parameters were measured *viz*. average roughness (S_a_, an amplitude parameter), texture aspect ratio (S_tr_, a spatial parameter) and developed interfacial area ratio (S_dr_, a hybrid parameter). MountainsMap^®^ Premium software (Digital Surf sarl, Besançon, France) was used for the measurement of the mentioned parameters. After topographical assessment of the surfaces, the same surfaces were qualitatively analyzed using a field-emission-gun scanning electron microscope (Feg-SEM, XL 30, Phillips, Eindhoven, The Netherlands).

### 3.4. The Proliferation of hMSC on Modified Ti Surfaces

The discs of the modified Ti surfaces (*n* = 6 per group) snuggly fit into 24-wells plates (Costar^®^, VWR, Haasrode, Belgium). Wells without discs, but with seeded cells, functioned as control (TCPS; control_4), while empty wells (without discs nor cells) were used to record the background signal. All discs were preconditioned for 24 h with Minimum Essential Medium Eagle, Alpha Modification (αMEM, Sigma, Bornem, Belgium). Prior to cell seeding, the surfaces were rinsed (3×) with PBS. Transfected cells, namely eGFP-fLuc-transfected hMSCs (see Supplementary Information) were used. Henceforth, the cells are designated as “hMSCs” for simplicity until and unless specified otherwise. hMSCs were seeded onto 3 (out of 6) discs of each group at a density of 3 × 10^3^ cm^−2^. The remaining half served as negative control. Bioluminescence image acquisition was performed at 1, 2, 3, 7, 11, 15 and 18 days post-seeding. Culture medium was removed, cells were washed with PBS, and D-luciferin at a concentration of 200 µg/mL dissolved in phenol red free culture medium was added. Imaging was performed at a standardized time point post-d-Luciferin addition. The mean bioluminescence imaging (BLI) signal (see Supplementary Information) of the negative control, as well as of the background noise was subtracted from experimental BLI signal values. BLI signal recorded at day 1 represented initial cell adhesion to the surfaces. BLI signals at subsequent time points were normalized to the day 1 signal in order to track the cell proliferation. After each acquisition, the wells were washed twice and the medium was added. All cultures were maintained at 37 °C in 95% humidified air and 5% CO_2_. At the endpoint, fluorescence microscope (Olympus, Tokyo, Japan) images of the cells were taken.

### 3.5. The Osteogenic Differentiation of hMSC on Modified Ti Surface

#### 3.5.1. Cell Culture Conditions

The discs with different surface treatments (*n* = 3 per group), positioned in 24-well plates, were preconditioned for 24 h in αMEM. Besides, non-transfected cells seeded on TCPS (NT-hMSCs/TCPS) were used as an additional control of the differentiation potential of transfected cells on TCPS (hMSC/TCPS). The cells were seeded at a density of 3 × 103 cells cm^−2^ and allowed to adhere for 24 h. Then, the medium was changed to osteogenic medium consisting of αMEM with 10% fetal bovine serum (FBS; PAA Laboratories GmbH, Pasching, Austria), 1% antibiotic-antimycotic (Gibco^®^ 15240, Life Technologies SAS, Saint Aubin, France), 0.292 g/L l-glutamine, 10 µM dexamethasone, 150 mM ascorbic acid and 2 mM β-glycerophosphate (all from Sigma, Bornem, Belgium). The surface mediated effect on osteogenic differentiation was evaluated at 0, 7, 14 and 21 days of induction. At each medium change, the culture medium was recovered and stored at −20 °C for enzyme linked immunosorbent assay (ELISA) analysis. For the determination of total DNA content and alkaline phosphatase activity in the cell layer at each time point, the surfaces were washed twice with PBS. The cell layer was detached from the surfaces by repeated (2×) trypsinization (trypsin/EDTA, Gibco^®^, Life Technologies) and shaking on a vibration platform for 60 s. The cells were separated from the solution by centrifugation (1500 rpm, 5 min), and 100 µL of lysis buffer (100 mM Na_2_CO_3_, 100 mM NaHCO_3_, 1 mM MgCl_2_) was added. All the procedures were carried out on ice. The cell lysates were stored at −80 °C till further analysis. The whole experiment was repeated twice.

#### 3.5.2. DNA Content

Double-stranded DNA content of the lysates of 0, 7, 14 and 21 days of differentiation was determined using a Quant-iT™ PicoGreen^®^ dsDNA assay Kit (Invitrogen, Frederick, MD, USA), according to the manufacturer’s instructions using a micro-plate reader (Infinite 200, Tecan, Mannedorf, Switzerland) at an emission/excitation wavelength of 480/520 nm. DNA content was determined from the calibration curve for DNA concentration range of 0 to 1000 ng/mL.

#### 3.5.3. ALP Activity

Alkaline phosphatase (ALP) activity of the lysates was determined by measuring enzymatic conversion of *p*-nitrophenyl phosphate (*p*NPP) to *p*-nitrophenol (*p*NP) due to ALP. Three freezing-thawing cycles were performed, after which, cellular debris was removed by centrifugation. The reaction mixture contained 50 µL of cell lysate, 250 µL of 2-amino 2-methyl 1-propanol (AMP) buffer (1.5 M) and 250 µL of *p*NPP (5.57 mg/mL, Sigma, Bornem, Belgium) in a final volume of 550 µL. After incubation at 37 °C for 1/2 h, the reaction was terminated by adding 1 mL of 0.1 M NaOH. *p*NP levels were measured at 410 nm.

#### 3.5.4. Enzyme-Linked Immunosorbent Assays (ELISA) of Cells’ Supernatants

In order to determine osteocalcin (OC), osteoprotegerin (OPG), vascular endothelial growth factor A (VEGF-A) and bone morphogenetic protein 2 (BMP-2) in the cell culture supernatants, ELISA tests were carried out. Cell culture supernatants after 0, 7, 14 and 21 days of differentiation were used for the determination of the first three markers, while measurement of BMP-2 was carried out for the supernatants of the relevant groups up to 14 days. Commercially available ELISA kits for OC, OPG, VEGF-A and BMP-2 were purchased from Invitrogen (Gent, Belgium), Ray BioTech (supplied by tebu-bio nv, Boechout, Belgium), Thermo-scientific (Erembodegem (Aalst), Belgium) and R&D systems (Minneapolis, MN, USA), respectively, and were used according to the manufacturer’s instructions. For determination of BMP-2 release profile, implants with adsorbed BMP-2 (n = 2) were placed in a 24-well plate with 1 mL DMEM (Sigma, Bornem, Belgium) at 37 °C. The medium was collected after 0, 1, 4, 7 and 14 days and stored at −20 °C till combined protein quantification. At each time point, the medium in the wells was replaced by fresh medium for monitoring cumulative release.

### 3.6. Statistical Analysis

Data were analyzed by analysis of variance, and significant differences between groups were determined using the Student’s *t*-test. Statistical significance was set at *p* < 0.05 for ALP and *p* < 0.02 for other comparisons.

## 4. Conclusions

In addition to the well-characterized and widely used uncoated Ti implants, amorphous microporous silica is suggested to be an excellent coating material for implants, which can be used as a delivery vehicle for bone morphogenetic protein 2. Adsorption of growth factors, such as BMP-2, onto AMS was found not only to accelerate osteogenic differentiation of progenitor cells, but also to create a suitable environment for further bone tissue development. In spite of the promising physical and chemical characteristics of bio-active glass and porous titanium surfaces reported in the literature, these surface coatings did not support cell viability and proliferation. In conclusion, functionalized AMS has a great though untapped potential for accelerating and enhancing bone regeneration and needs further exploration in the implant osseointegration and tissue regeneration fields.

## Supplementary Materials

Supplementary File 1
