# Supplementary material for: Modified Titanium Surface-Mediated Effects on Human Bone Marrow Stromal Cell Response
Source: Materials (Basel). 2013 Nov 28;6(12):5533–48. doi: 10.3390/ma6125533 (PMC5452737; doi:10.3390/ma6125533)
Supplement: Supplementary File 1 [file materials-06-05533-s001.pdf]

Supplementary Information

1. Experimental Section

1.1. Isolation and Culture of Bone Marrow-Derived hMSCs

Human bone marrow-derived MSCs (hMSCs) were isolated from femoral heads of 5 donor patients undergoing total hip replacement surgery, using a procedure previously described [1,2]. Whole bone marrow was curetted from interior of femoral neck and head, washed in Minimum Essential Medium Eagle—Alpha Modification (αMEM, Sigma, Bornem, Belgium) and separated from trabecular bone fragments and other tissues using an 18-gauge needle attached to a 25-mL syringe. The bone marrow cells were centrifuged at 1200 rpm for 5 min to separate them from residual adipose tissue and resuspended in Hank's balanced salt solution (HBSS; Fisher Scientific, Illkirch, France). Mono-nucleated cells were isolated via Ficoll density centrifugation at 600 g for 30 min. Cells were then resuspended in αMEM with 0.292 g/L L-glutamine (G7513, Sigma, Bornem, Belgium), supplemented with 10% fetal bovine serum (FBS; PAA Laboratories GmbH, Pasching, Austria) and 1% antibiotic-antimycotic (Gibco® 15240, Life Technologies SAS, Saint Aubin, France), and plated in 75-cm^2 cell culture flasks. Cells were maintained O/N at 37 °C in a humidified environment with 5% CO_2. Following day, culture medium was changed to remove non-adherent cells. Subsequent medium changes were made every 3 days. Upon 80% confluence, the cells were detached using 0.05% trypsin/EDTA (Gibco®, Life Technologies) and cryo-preserved in FBS containing 10% dimethyl sulfoxide (DMSO, D2650, Sigma, Bornem, Belgium). Cells from 5 different donors were thawed, pooled and expanded for 2 passages prior to experimentation. Same passage number (P5) was used for all experiments.

1.2. hMSC Viral Transfection

hMSCs were genetically modified in one single gene transfer step using a double reporter, *i.e.*, eGFP (enhanced Green Fluorescent Protein) and fLuc (firefly Luciferase) lentiviral vector [3]. hMSCs were plated at density of 8×10^3 cells/cm^2 and cultured under standard cell culture conditions for 24 h. The cells were then rinsed twice with phosphate buffered saline (PBS), infected with 2×10^6 viral particles/cm^2 (250 particles/cell) added to complete αMEM medium and cultured again under standard conditions for 24 h. After 2 medium changes over 48 h, cells were observed by fluorescence microscope and further expanded. At confluence, cells were detached by trypsinization and characterized.

1.3. hMSC Characterization by Flow Cytometry

1.3.1. Phenotypic Analysis

hMSCs were phenotypically characterized by fluorescence-activated cell sorting (FACS) according to stipulated criterion for hMSCs [4]. FACS analysis (Guava easyCyte, Millipore, Molsheim, France) was performed using Phycoerythrin (PE)-conjugated CD45, CD73 and CD105 antibodies (Immunotools, Friesoythe, Germany) to confirm that the phenotype of both non-transfected and transfected hMSCs was maintained after expansion in the culture. PE-conjugated isotype matched negative control antibodies were added. Fixed hMSCs (prepared from trypsinized monolayers at passage 6) were

incubated with antibodies against each surface marker for 45 min at 4 ℃. Cells were resuspended in PBS with 1% bovine serum albumin (BSA) and analyzed using Cytosoft software (Millipore).

1.3.2. Proliferation Potential Analysis

The proliferation of non-transfected *versus* transfected hMSCs was evaluated by seeding the cells (5×10^3 cells/cm^2) in 24 well plates (n = 3) in basic cell culture medium (αMEM with 10% FBS, 1% antibiotic and anti-mycotic and L-Glutamine at 0.292 g/L; all from Sigma, Bornem, Belgium). Cell numbers were determined after 1, 3 and 6 days of cell seeding by FACS on fixated samples.

1.4. Bioluminescence Imaging for Monitoring Cell Proliferation

For the purpose of cell proliferation evaluation by bioluminescence imaging (BLI) [5], determination of the correlation between the cell number and the bioluminescent intensity signal was performed prior to experimentation with modified titanium surfaces. Cell suspensions at serial dilutions were added to a black 96-wells plate at densities between 0 and 5×10^5 cells/well. D-luciferin solution (Promega, Leiden, The Netherlands) at a concentration of 200 µg/mL was added and bioluminescence images were acquired by IVIS100 optical imaging system (Xenogen Corporation, Alameda, CA, USA) with 2 s exposure time and small binning setting. BLI (expressed in # photons/s for a defined area) was determined with the help of LivingImage® software (version 2.50.1, Xenogen Corporation). The experiment was carried out in duplicate. Background signal was subtracted from measured BLI signal and obtained value was plotted against the number of cells.

2. Results and Discussion

2.1. hMSC Characterization

2.1.1. hMSC Phenotype

Figure S1 illustrates phenotypic characterization results for non-transfected and eGFP-fLuc-transfected hMSCs. FACS analysis showed that the cells were negative for CD45 expression and positive for CD73 and CD105, a phenotype characteristic for hMSCs. Viral cell transfection did not alter the expression of these surface markers.

Figure S1. Flow cytometric analyses for CD45, CD73 and CD105 surface marker expressions for (**a**) non-transfected (NT-hMSC) and (**b**) transfected (eGFP-fLuc-hMSC). Gray line indicates the control of CD marker isotypes.

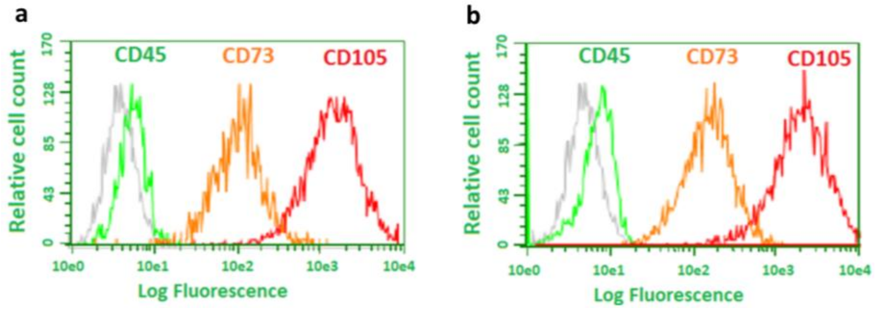

2.1.2. Efficacy of Viral Transfection—Proliferation Potential

An efficacy of viral transfection of up to 60% was recorded, as determined by FACS. The transfection ratio remained constant during cell proliferation (as displayed in Figure S2a) as well as over different passages (results not shown). Furthermore, no differences could be observed in cell proliferation rate of both cell groups (Figure S2b).

Figure S2. Assessment of the efficacy of lentivirus-mediated cell transfection. (**a**) Stability of the transfected cells during proliferation; (**b**) Comparison of the proliferation rate of non-transfected (NT-hMSC) and transfected (eGFP-fLuc-hMSC) cells.

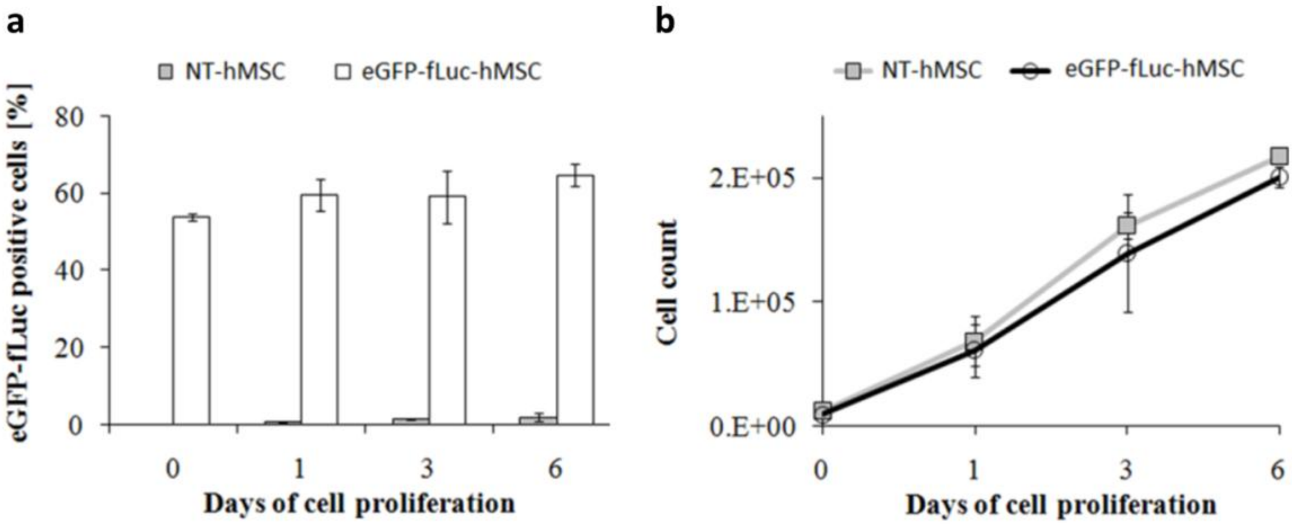

Human MSCs were selected for use in the *in vitro* cell culture experiments. In order to prove the cells' nature, surface marker expression verification for hMSCs [4] was carried out. Furthermore, it was shown that lentiviral transfection did not alter phenotypic expressions of isolated cells, neither their proliferation behavior. Therefore, eGFP-fLuc transfected human bone marrow derived stromal cells were considered appropriate for evaluation of the biocompatibility of specific modified Ti surfaces and for exploration of their pro-osteogenic properties.

2.2. Bioluminescence Imaging for Monitoring Cell Proliferation

In order to use BLI as a tool to monitor cell proliferation, a correlation between BLI signal and actual cell number was checked. The correlation between emitted signal and cell number was found to be linear (R^2 = 0.996 and R^2 = 0.995 for duplicate experiments) (Figure S3). The linear correlation between cell number and BLI signal indicate that BLI method was suitable for monitoring cell proliferation on modified Ti surfaces.

Figure S3. Correlation between the bioluminescent signal of eGFP-fLuc-hMSCs and the cell number. (Experiment in duplicate).

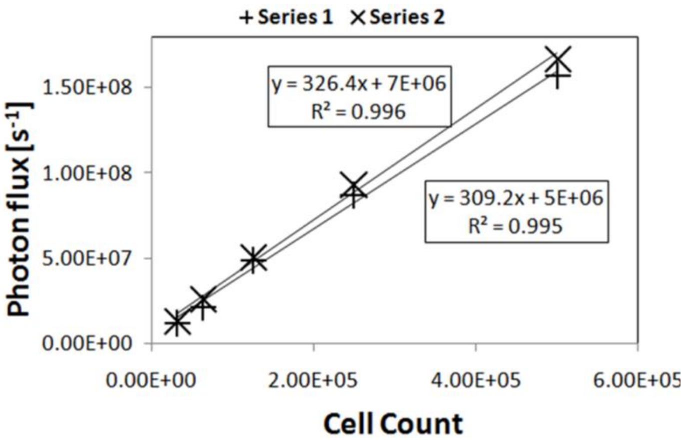

References

1. Pittenger, M.F.; Mackay, A.M.; Beck, S.C.; Jaiswal, R.K.; Douglas, R.; Mosca, J.D.; Moorman, M.A.; Simonetti, D.W.; Craig, S.; Marshak, D.R. Multilineage potential of adult human mesenchymal stem cells. *Science* **1999**, *284*, 143–147.
2. Friedenstein, A.J.; Chailakhjan, R.K.; Lalykina, K.S. The development of fibroblast colonies in monolayer cultures of guinea-pig bone marrow and spleen cells. *Cell Tissue Kinet.* **1970**, *3*, 393–403.
3. Ibrahimi, A.; Vande Velde, G.; Reumers, V.; Toelen, J.; Thiry, I.; Vandeputte, C.; Vets, S.; Deroose, C.; Bormans, G.; Baekelandt, V.; *et al*. Highly efficient multicistronic lentiviral vectors with peptide 2A sequences. *Hum.Gene Ther.* **2009**, *20*, 845–860.
4. Dominici, M.; Le Blanc, K.; Mueller, I.; Slaper-Cortenbach, I.; Marini, F.; Krause, D.; Deans, R.; Keating, A.; Prockop, D.; Horwitz, E. Minimal criteria for defining multipotent mesenchymal stromal cells. The International Society for Cellular Therapy position statement. *Cytotherapy* **2006**, *8*, 315–317.
5. Logeart-Avramoglou, D.; Oudina, K.; Bourguignon, M.; Delpierre, L.; Nicola, M.A.; Bensidhoum, M.; Arnaud, E.; Petite, H. *In vitro* and *in vivo* bioluminescent quantification of viable stem cells in engineered constructs. *Tissue Eng. Methods* **2010**, *16*, 447–458.

© 2013 by the authors; licensee MDPI, Basel, Switzerland. This article is an open access article distributed under the terms and conditions of the Creative Commons Attribution license (http://creativecommons.org/licenses/by/3.0/).
